# Enhancing Ammonia Concentration Prediction with a Transfer-Learning-Based Model: Application in a Pig Farm

**DOI:** 10.3390/ani16040609

**Published:** 2026-02-14

**Authors:** Sunhyoung Lee, Rack-Woo Kim, Hakjong Shin, Sang-Shin Lee, Won-Gi Choi

**Affiliations:** 1 Department of Agriculture Engineering, College of Industrial Sciences, Kongju National University, 54 Daehak-ro, Yesan-eup, Yesan-gun 32439, Chungcheongnam-do, Republic of Korea; dltjsgud2@smail.kongju.ac.kr; 2 Department of Smart-Farm Engineering, College of Industrial Sciences, Kongju National University, 54 Daehak-ro, Yesan-eup, Yesan-gun 32439, Chungcheongnam-do, Republic of Korea; 3 Agriculture, Animal, Aquaculture & Ocean Intelligence Research Center, Electronics and Telecommunications Research Institute, 218 Gajeong-ro, Yuseong-gu, Daejeon-si 34129, Republic of Korea; hakjong@etri.re.kr; 4 Autonomous Intelligent System Research Center, 25 Saenari-ro, Bundang-gu, Seongnam-si 13509, Gyeonggi-do, Republic of Korea; sslee@keti.re.kr (S.-S.L.); cwk1412@keti.re.kr (W.-G.C.)

**Keywords:** artificial intelligence, ammonia, transfer learning, XGBoost, swine house

## Abstract

Globally, the swine industry is a major component of agricultural production, and the increasing scale and intensification of pig farming have heightened concerns about NH_3_ emissions. As farms expand and adopt smart farming technologies, there is a need for reliable prediction of NH_3_ concentrations without relying solely on costly physical sensors. In this study, we developed an artificial intelligence-based prediction model for NH_3_ concentration in commercial pig houses and examined the effects of data collection intervals and learning strategies. We compared a standalone model trained only on local data with a transfer learning model that adapts a pre-trained model to a target farm with limited data. Transfer learning consistently outperformed the standalone approach across all data collection intervals (10, 20, 30 and 60 min). The best-performing Random Forest and XGBoost models achieved a coefficient of determination (*R*^2^) of 0.969, root mean square error (*RMSE*) of about 1.0 ppm and mean absolute percentage error (*MAPE*) below 5%. These results show that transfer learning can provide accurate NH_3_ predictions in swine housing even with sparse data, supporting more sustainable and data-efficient environmental management.

## Simple Summary

Pig farms around the world are becoming larger and more intensive. This can lead to high levels of ammonia gas, which can adversely affect the health of pigs and workers and cause strong odours around farms. Measuring this gas with physical sensors at every farm is costly and can be difficult to maintain. In this study, we built a computer-based model to predict ammonia concentrations using data collected at commercial pig farms, including the number and weight of pigs, indoor temperature and humidity, and the rate of air exchange. The model was first trained on detailed data from one farm and then tested for its applicability to another farm with only a small amount of data. The adapted model predicted ammonia concentrations more accurately than a model trained only on the smaller local dataset. This indicates that farms with limited data can still obtain accurate predictions of harmful gas levels by using knowledge learned from other farms. Such tools can help farmers improve animal welfare, protect workers and nearby residents, and support more sustainable and environmentally friendly pig production.

## 1. Introduction

### 1.1. Background

Globally, the livestock industry contributes approximately 40% of agricultural value added and supports the livelihoods and food security of more than one-sixth of the world’s population [1]. Pork production has increased by approximately 140% since the 1960s, exceeding 113 million tonnes of global consumption in 2022, while the global pig population has approached one billion [2,3,4]. To increase productivity under spatial constraints, producers have expanded housing infrastructure and adopted smart livestock systems, resulting in fewer farms but larger herd sizes per farm [5,6,7].

With increasing production intensity, enclosed mechanically ventilated housing has become widespread to improve climate control, biosecurity, and management efficiency. However, gases and particulates generated from animals and manure can accumulate under inadequate or poorly managed ventilation, degrading indoor air quality. Among these emissions, NH_3_ contributes to secondary particulate formation and irritates the eyes and respiratory mucosa of animals and workers, with potential impacts on surrounding ecosystems. Poor control of the in-house gaseous environment can compromise animal welfare and productivity, while emissions released outdoors may cause odour nuisance and environmental concerns [8,9,10,11,12].

Therefore, accurately measuring and predicting NH_3_ concentrations in pig housing facilities is of critical importance. However, the installation and maintenance of NH_3_ sensors incurs significant financial and operational burdens, necessitating the use of data-driven approaches for predicting indoor NH_3_ concentrations. Several studies have measured NH_3_ levels within pig facilities, including direct measurements using emission estimation methodologies [13,14,15]. Recent research has begun to apply artificial intelligence models to predict NH_3_ emissions [16,17,18,19,20,21]. Previous studies have approached NH_3_ concentration prediction using various methods, including simulation modelling, field-based measurements, physicochemical emission estimation, and AI-driven predictive modelling.

These models have primarily been developed using data collected directly from individual target facilities [22,23,24], with machine learning (ML) and artificial intelligence techniques employed to account for the relationships between diverse environmental variables. Although a wide array of AI-based predictive models is now being applied in the livestock sector, these approaches require model development based on site-specific, on-farm data. However, field-based research entails long-term and high-cost data collection processes, and securing large-scale, high-quality datasets that reflect diverse operational conditions is difficult. Although large amounts of high-quality training data are essential to ensure the accurate performance of ML-based models, such conditions are rarely met in real-world livestock production environments outside laboratory settings. As a potential solution to these limitations, transfer learning, supported by advances in artificial intelligence technologies and computational capabilities, has recently gained attention as an approach that compensates for data scarcity and enhances the generalisability of predictive models.

### 1.2. Review of Previous Studies

Transfer learning is a technique that enables the application of knowledge or feature representations acquired from a source domain to a target domain, thereby achieving high predictive performance even with limited data. This approach has been widely utilised in various fields, including natural language processing, image recognition, and medical data analysis. By leveraging knowledge learned from a source domain, transfer learning reduces the amount of data needed in the target domain and enables rapid adaptation via fine-tuning. This is particularly relevant for livestock facilities, where sensor installation and long-term measurements are often constrained by cost and operational feasibility. In the context of livestock environmental prediction, transfer learning is gaining increasing attention because of its advantages in reducing data collection costs and enhancing model reusability. Particularly in situations where training data are scarce, models developed through transfer learning have been recognised as practical solutions because they maintain high generalisation performance while remaining adaptable to diverse environmental conditions. This section examines existing research on transfer learning techniques and predictive models for NH_3_ concentrations within livestock facilities. Based on this review, the feasibility of applying transfer learning to predict NH_3_ concentrations in environments with limited ammonia-related datasets was explored.

#### 1.2.1. Studies on Transfer Learning Models

Park et al. (2022) developed a high-performance model for predicting the energy consumption in buildings of the same type by leveraging a pretrained model, even under conditions in which the dataset for the target building was insufficient [25]. Park et al. (2023) conducted a study targeting built environments by applying a hybrid ensemble transfer learning approach to predict natural ventilation using a small set of environmental data collected through field experiments [26]. Sharma et al. (2024) conducted a comparative analysis of various transfer learning methods to experimentally evaluate the most effective techniques for predicting building energy usage [27]. Jiang and Lee (2019) proposed a deep-learning-based transfer learning framework for predicting indoor temperature dynamics in smart buildings by applying a pre-trained thermal dynamics model to buildings with different structures and operational conditions [28]. Hossen et al. (2025) systematically reviewed the conceptual application of transfer learning to address data scarcity, along with its application cases across various agricultural domains [29]. Al Sahili and Awad (2022) proposed and evaluated a domain-specific pre-trained model for the fine-grained classification of agricultural images using transfer learning [30].

These transfer-learning-based predictive models have demonstrated the potential to achieve high predictive performance in data-scarce environments across a variety of fields. However, in the livestock sector—particularly within complex and irregular environments such as pig housing—research applying transfer learning techniques remains limited. In particular, virtually no studies have applied transfer learning utilising a high-quality, pre-trained source domain model to predict key environmental indicators, such as NH_3_ concentration, in target facilities where data are insufficient. NH_3_ concentration in pig farms is a critical variable that is directly linked to animal health, worker safety, odour control, and air pollution, making its prediction an essential requirement for sustainable farm management. Nevertheless, in real farming conditions, sensor degradation, environmental fluctuations, data loss, and noise often result in low-quality data, which has significantly limited the research efforts on NH_3_ concentration prediction using transfer learning models.

#### 1.2.2. Prediction Models for Livestock Housing Environments

Although no existing studies have directly applied transfer learning to NH_3_ concentration prediction models in livestock facilities, studies employing ML and deep learning techniques have been actively conducted. Peng et al. (2023) developed an NH_3_ concentration prediction model using environmental data collected from pig farms and compared and validated the predictive performance of different algorithm types [31]. The model optimised using particle swarm optimisation exhibited superior predictive accuracy. Yeo et al. (2023) developed and evaluated ML-based regression models to predict the internal temperature and CO_2_ concentration in mechanically ventilated pig barns and found that the random forest regression model demonstrated the highest predictive performance [32]. Lee et al. (2022) constructed a long short-term memory (LSTM)-based prediction model using data collected from both mechanically and naturally ventilated duck houses to predict internal environmental conditions and compared the model performance across seasons and ventilation types [33]. Kim et al. (2023) addressed the challenge of long-term environmental data scarcity in fattening pig houses by developing an LSTM-based recurrent neural network model to predict missing internal temperature data. Their experiments confirmed that the model can correct missing values with an error rate of less than 3.5% [34].

### 1.3. Scope and Objectives of the Study

This study proposes a transfer learning framework for predicting NH_3_ concentrations in mechanically ventilated pig houses by adapting an XGBoost model pre-trained using data from a standardised pig facility. Using routinely monitored indoor environmental and management variables, the framework is designed to reflect practical conditions in which continuous NH_3_ sensing may be unavailable; therefore, this study predicts NH_3_ concentrations using these variables excluding NH_3_ concentration as an input feature. Although NH_3_ was measured during the field campaigns, it was used only as the prediction target (label) for model training and independent evaluation. Specifically, this study aims to develop a source-domain pre-trained model, evaluate its transferability to a target farm under limited target-domain NH_3_-labelled data by comparison with a standalone target-trained model, assess robustness across different sampling intervals and operating conditions, and identify key factors associated with NH_3_ variability to support practical monitoring and control.

## 2. Materials and Methods

**Figure 1:**
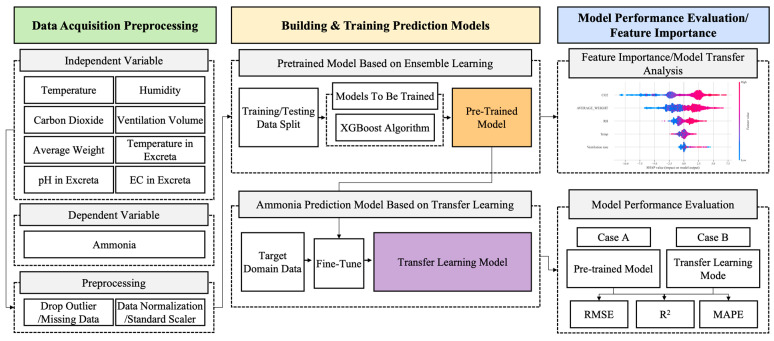
Schematic for the development of the ammonia-prediction model. Abbreviations: electrical conductivity (EC).

Figure 1 illustrates the procedural framework of this study, which aimed to develop a reliable NH_3_ concentration prediction model by transferring pre-trained knowledge from a source domain to a target domain with differing climatic conditions and facility structures. Initially, a pre-trained model was developed using empirical data collected from a facility designated as the source domain. An extreme gradient boosting (XGBoost) algorithm, which is a decision-tree-based ensemble learning method, was employed for this purpose. Subsequently, the fine-tuned features extracted from the pre-trained model were used as input variables to predict the NH_3_ concentrations in the target domain. The predictive performance of two modelling approaches was then evaluated and compared: one based on a standalone XGBoost model and the other on a transfer-learning-based model.

### 2.1. Structural Characteristics of Target Facilities and Data Collection Environment

**Figure 2:**
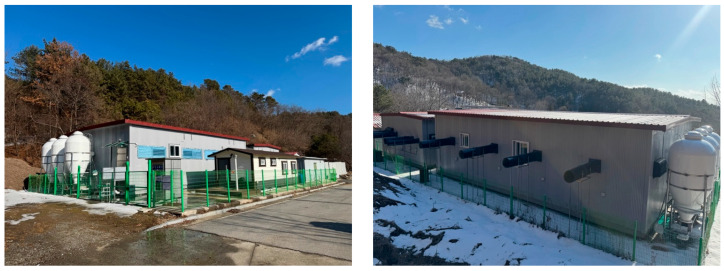

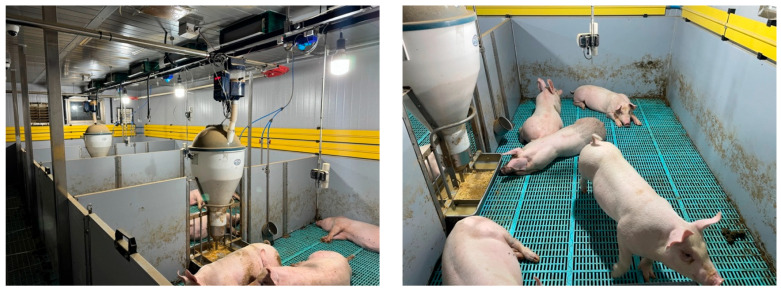
Source Domain: Small-scale smart pig farming engineering and demonstration facility.

To develop an indoor NH_3_ concentration prediction model and apply transfer learning, field experiments were conducted at two pig farms with different data collection periods and structural characteristics. The source facility used for pre-training was located at 1 Galsin-ri, Daheung-myeon, Yesan-gun, Chungcheongnam-do, South Korea (36.6685° N, 126.8657° E), within the engineering research complex of Kongju National University’s Yesan Campus, as shown in Figure 2. This facility consists of a nursery, two growing–finishing rooms, and a fattening barn. During the experimental period, 24 early-stage fattening pigs (aged 16–20 weeks) were housed. The body weight of the pigs increased from approximately 59.77 to 81.25 kg over the course of the study. The facility features a ceiling space of 1.5 metres above the pens, through which outside air enters via the corridor, flows into the ceiling space, and is discharged through ceiling slots by two exhaust fans (COCO-400C, Dongsung COCO Fan), constituting its ventilation system.

**Figure 3:**
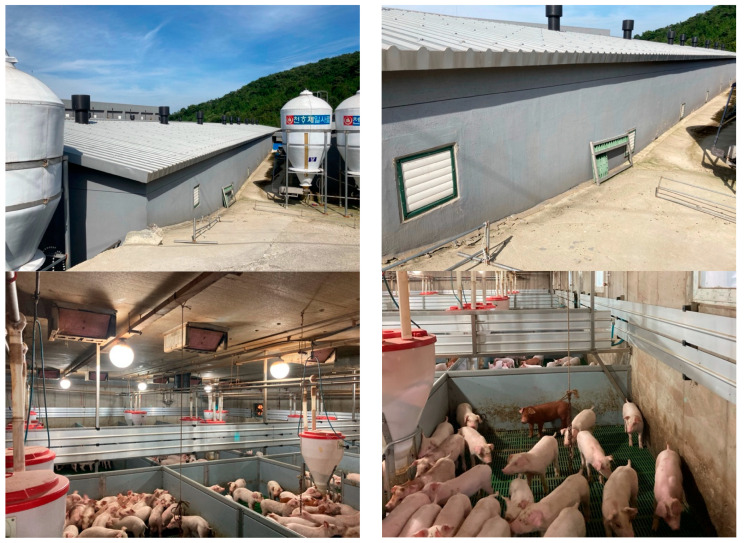
Target Domain: Eco farm for applying an ammonia prediction model using transfer learning.

The target facility to which transfer learning was applied was Eco Farm, located at 956-1 Changnyeong-ri, Nakan-myeon, Suncheon-si, Jeollanam-do, South Korea (34.9551° N, 127.3689° E), as shown in Figure 3. This facility consists of two growing pig houses, each housing approximately 900 pigs for 7–10 weeks. During this period, the pigs’ body weight increased from approximately 7 to 25 kg. The facility employs a chimney-type exhaust fan system to ventilate the indoor air into the outside environment.

Although the two facilities differ in aspects such as regional climate, production scale, and structural layout, they share common features in terms of ventilation approach and the availability of core environmental variables, enabling cross-facility model adaptation. NH_3_ measurements were collected for ground-truth validation but were not included among the model input features. These similarities provide suitable conditions for evaluating the applicability and generalisability of transfer-learning-based predictive models. Therefore, in this study, the Kongju National University facility was designated as the source domain and Eco Farm as the target domain. Prediction models were developed using the data collected from both facilities, and the applicability and performance of transfer learning were evaluated empirically.

Additionally, to reflect the actual operating conditions, no artificial restrictions were placed on access or routine management activities within either facility. During the summer experiments, cooling systems were activated, and during the winter experiments, heating systems were used to accommodate seasonal temperature variations.

#### 2.1.1. Pre-Training Model Dataset

The factors influencing NH_3_ emissions from pig housing were identified based on the findings of previous studies and technical reports [35,36]. Accordingly, various internal and external environmental variables, manure characteristics, and biological information were selected for data collection (Table 1). To gather the necessary data, livestock environmental sensors (DOL, Aarhus, Denmark, HMdigital Co., Ltd., Seoul, Korea and Korea Industrial Machinery Co., Ltd., Seoul, Korea) were installed to measure the indoor temperature and relative humidity (RH); CO_2_, NH_3_, and methane (CH_4_) concentrations; manure temperature; pH; and electrical conductivity. Although NH_3_ concentration was measured during the campaign, it was used only as the prediction target (label) and was not included among the input features.

**Figure 4:**
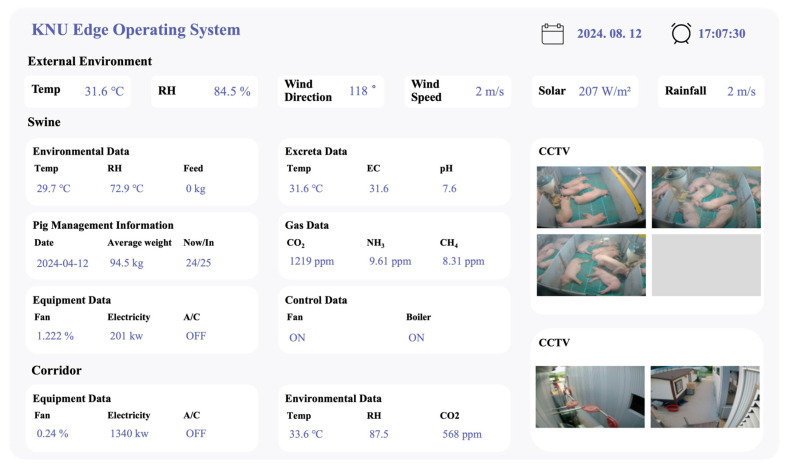
Web browser-based testbed monitoring system.

The training dataset for the predictive model was collected using the oneM2M system, an international standardisation initiative designed to ensure compatibility within the increasingly complex and diverse Internet of Things (IoT) market. This system supports the development of various IoT protocols, provides network functionalities that comply with international standards, and offers management capabilities for both data and devices [37]. Data were measured at 5 min intervals from 23 July to 31 August 2024, using the installed specification management and environmental sensor devices. The data were transmitted via receivers to a network-connected storage unit, enabling users to monitor the data in real time through a web interface (Figure 4). The stored data were processed using Microsoft Excel for training. During data collection, outliers were screened on a per-variable basis using the 1.5 × IQR rule. Values below the first quartile minus 1.5 times the interquartile range or above the third quartile plus 1.5 times the interquartile range were removed. Missing values were then imputed using mean values calculated within the corresponding categorical groups. The monitoring interval for NH_3_-related influencing factors and measured parameters within the small-scale testbed was determined based on the required data volume for designing NH_3_ emission prediction algorithms derived from prior studies and related literature [35,36], as well as the storage capacity of the existing database.

#### 2.1.2. Transfer Learning Model Training Data

The dataset used to construct the transfer-learning-based predictive model included internal environmental factors and biological information collected at 5 min intervals and stored in a real-time database (Table 2 and Table 3). The key variables gathered for model training were indoor temperature, relative humidity (RH), CO_2_ concentration, NH_3_ concentration, ventilation rate, and average body weight of the pigs. These served as the primary independent variables for the model development. Various sensors were installed throughout the facility to obtain high-precision environmental data. Temperature, RH, and CO_2_ levels were measured using an integrated sensor SH-VT260VS (Soha Tech, Seoul, Korea), whereas NH_3_ concentration was measured in duplicate using GV-110 (GASTEC, Ayase, Japan) and MultiRAE IR (RAE Systems, San jose, CA, USA) sensors. The ventilation rate was calculated using a differential pressure sensor (TSI Inc., Shoreview, MN, USA). All collected data were stored and managed in real time using a HeidiSQL-based database system. Time synchronisation was conducted by aligning all sensor measurements to a common 5 min time grid (the original sampling interval). Missing entries were filled by linear interpolation, using the mean of the nearest values before and after the missing time point. The synchronised 5 min dataset was then sampled to create datasets at 10, 20, 30, and 60 min intervals for subsequent analyses. This high-resolution dataset formed the basis of the input variable set for transfer learning and contributed to the improved predictive performance of the model.

The data were collected at 5 min intervals from 10 January to 5 February 2022, using installed specification management and environmental sensor devices. The collected data were normalised and used for model training. To prevent unnecessary duplications during data acquisition, duplicate entries were removed using C language-based string-processing functions. In addition, because data collection intervals can vary between sensors, time synchronisation was performed across all datasets to ensure a consistent and coherent time-series structure.

### 2.2. ML Model

**Figure 5:**
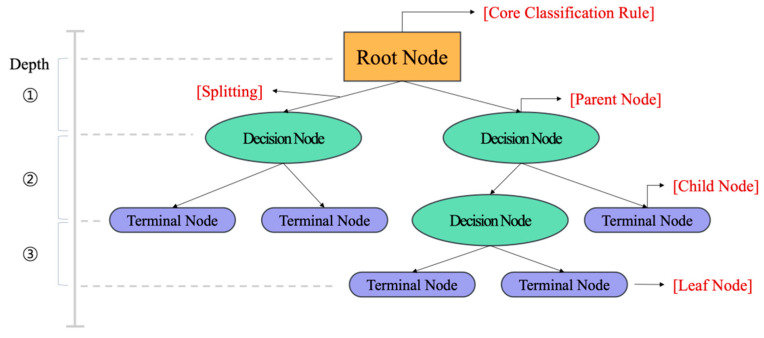
Ensemble learning using a decision tree.

Among ML-based predictive models, the ensemble learning technique enhances predictive performance by combining multiple weak learners, typically constructed using decision trees (Figure 5). Three primary ensemble methods are available. The voting method integrates the prediction results from different algorithms by averaging or majority voting. Second, the bagging method generates multiple subdatasets through bootstrapped sampling from the full dataset, trains models in parallel using the same algorithm, and aggregates the results by averaging or majority voting. Third, the boosting method sequentially trains models by incorporating the errors of previous learners to incrementally improve prediction accuracy. These ensemble techniques offer superior generalisation performance compared with single models and are robust against noise and outliers, making them effective tools for prediction in complex livestock environments. In this study, we used XGBoost, a boosting-based ensemble algorithm, as the final predictive model rather than a bagging-based ensemble.

#### 2.2.1. XGBoost

**Figure 6:**
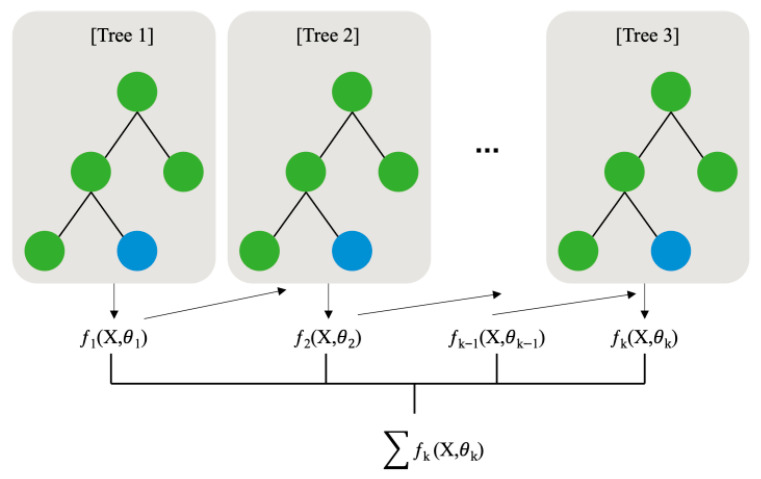
XGBoost algorithm.

To predict NH_3_ concentrations, this study employed XGBoost, a boosting-based ensemble learning technique within the family of ML-based predictive models. XGBoost is an algorithm that enhances predictive performance by sequentially combining multiple weak learners, typically regression trees. Each tree was trained to correct the prediction errors of the previous tree, and the overall model was constructed through repeated residual prediction processes (Figure 6). During training, the model was optimised to minimise the objective function using gradient descent, which allows for superior computational efficiency and prediction accuracy compared to conventional gradient boosting machine methods [38]. XGBoost, implemented as an open-source library, offers high scalability and flexibility and can be applied to both classification and regression tasks [39]. In this study, the characteristics of XGBoost were leveraged using various internal environmental variables collected from farm settings as input features and employed to predict indoor NH_3_ concentrations within a livestock facility.

#### 2.2.2. Generalization of the Transfer-Learning-Based Model

In tree-based models, transfer learning is implemented by reusing a previously trained booster rather than transferring feature embeddings. In this study, we performed transfer learning in XGBoost by continuing boosting from a source-domain model. Specifically, an XGBoost model was first trained on the source-domain dataset and saved. For the target-domain training, we loaded the source booster as an initial model and added additional boosting rounds using the target-domain data, using the same feature set and hyperparameters to maintain model compatibility. In Python, this was implemented using xgboost.train() with the xgb_model argument to initialise training from the pre-trained booster, followed by training for N_target additional boosting rounds on the target dataset. 

In the case of pig housing facilities, considerable differences in internal environmental conditions arise owing to variations in structural design, ventilation systems, feed composition, stocking density, and environmental management practices across farms, all of which directly influence NH_3_ emissions. When such domain-level differences exist, applying a pre-trained predictive model without adaptation often results in degraded performance [23].

To address this issue, the generalisability of an NH_3_ concentration prediction model developed using data from the Kongju National University testbed was evaluated by applying it to a general commercial pig farm. For this purpose, a transfer learning approach was introduced to maintain high predictive performance even under differing farm conditions. Transfer learning is an ML-strategy that enhances prediction accuracy by effectively transferring the knowledge of a pre-trained model from a source domain to a target domain, particularly in environments where sufficient training data are difficult to obtain [40].

A relevant case in the agricultural domain was presented in a study by Esparza-Gómez et al. (2023), where data from an existing greenhouse were used to predict the temperature in a new greenhouse environment [39]. The transfer learning approach used in this study aligns with the trends in the field.

#### 2.2.3. Model Validation Method

To evaluate the performance of the predictive model in estimating specific factors, the performance metrics used were the coefficient of determination (*R*^2^), root mean square error (*RMSE*), and mean absolute percentage error (*MAPE*). *R*^2^ represents the proportion of variance in the dependent variable that can be explained by the independent variables, and ranges from zero to one. A value closer to one indicates that the model explains the data more effectively. *R*^2^ is defined as follows:

$$R^{2}=1-\frac{SS_{res}}{SS_{tot}}.$$

$SS_{res}:$ The sum of the squares of residuals is the sum of the squared differences between the actual values and the predicted values.

$SS_{tot}$: The total sum of the squares is the sum of the squared differences between the actual values and the mean value.

The *RMSE* is a key metric used to evaluate the predictive performance of regression models. It represents the average difference between the predicted and actual values; thus, a smaller *RMSE* value indicates that the model predictions are closer to the actual outcomes. The *RMSE* is defined as follows:

$$RMSE=\sqrt{\frac{1}{n}\sum_{i=1}^{n}{\left(y_{i}-{\hat{y}}_{i}\right)}^{2}},$$

where n is the number of samples, $y_{i}$ is the actual value, and ${\hat{y}}_{i}$ is the predicted value.

The *MAPE* is a metric used to evaluate the prediction accuracy of a regression model by expressing the error between predicted and actual values as a percentage. As the *MAPE* measures prediction error in relative terms, it is particularly useful for comparing datasets of different scales. Therefore, a lower *MAPE* value indicates that the model predictions are closer to the actual values. The *MAPE* is defined as follows:

$$MAPE=\frac{1}{n}\sum_{i=1}^{n}\left|\frac{y_{i}-{\hat{y}}_{i}}{y_{i}}\right|\times 100,$$

where n is the number of samples, $y_{i}$ is the actual value, and ${\hat{y}}_{i}$ is the predicted value.

### 2.3. Development of a Transfer-Learning-Based Predictive Model for Indoor NH_3_ Concentration in Livestock Facilities

#### 2.3.1. Pre-Trained Model

To perform transfer learning effectively, constructing a pre-trained model with high predictive accuracy is essential. In this study, an XGBoost-based pre-trained model was developed using the source-domain dataset. The dataset was recorded at 5 min resolution and was split chronologically into training and test periods (80%/20%) to avoid information leakage: the training period was 10 January 2022 09:40–31 January 2022 02:45, and the test period was 31 January 2022 02:50–05 February 2022 07:05. To prevent data leakage during preprocessing, resampling to the target sampling intervals (10, 20, 30, and 60 min) and interpolation of missing values were performed separately within each split using only the information available within the corresponding period.

The input variables for model training were selected through an importance analysis of the collected environmental and management data, thereby quantitatively incorporating the key factors influencing NH_3_ concentration. To improve performance, GridSearchCV was applied to optimise key hyperparameters, and the optimal combinations were identified (Table 4). To enhance generalisability and reduce overfitting, the maximum tree depth (max_depth) was set to 10 and the learning rate was set to 0.1. In addition, feature and instance sampling were applied during training to reduce sensitivity to specific data patterns.

#### 2.3.2. Transfer Learning Model

To evaluate the performance of the transfer-learning-based predictive model, the pre-trained XGBoost model developed on the source domain (Section 2.3.1) was transferred to the target domain using the transfer learning dataset. The target-domain dataset was recorded at 5 min resolution and was split chronologically into training and test periods (80%/20%): the training period was 23 July 2024 14:40–11 August 2024 16:35, and the test period was 11 August 2024 16:40–16 August 2024 11:10. As with the source domain, resampling and interpolation were conducted after the chronological split and applied separately within the training and test periods to prevent leakage.

Two experimental cases were designed to compare prediction accuracies and to examine the effects of sampling interval and limited target-domain training data. Case A trained standalone XGBoost regression models using only the target-domain data after resampling to 10, 20, 30, and 60 min intervals, and compared their performance across intervals. Case B implemented transfer learning by initialising the target-domain model with the source-domain pre-trained XGBoost model and then fine-tuning it using target-domain subsets corresponding to the same sampling intervals (10, 20, 30, and 60 min). Prior to fine-tuning, the input feature set was aligned to match the input variable configuration of the pre-trained model. Missing predictors in the target-domain dataset were replaced with zeros to maintain the same feature dimension. The amount of target-domain training data used for adaptation was adjusted according to the fine-tuning ratio. Prediction performance was then evaluated on an independent evaluation set using *R*^2^, *RMSE*, and *MAPE*.

### 2.4. SHAP-Based Analysis of Feature Importance and Model Performance in Transfer Learning

Explainable artificial intelligence (XAI) techniques have been increasingly employed to interpret the relationship between the outputs of a predictive model and its input variables. These methods enable model developers to understand the contribution of each variable and are considered essential for adjusting model structure and enhancing interpretability.

Among the various approaches used to ensure model explainability, *SHAP* has recently gained wide adoption as a method for quantitatively evaluating the contribution of each input feature to a model’s predictions. *SHAP* expresses the influence of individual input variables on model predictions in a numerically interpretable form and is particularly effective for interpreting tree-based models.

*SHAP* is based on the Shapley value from cooperative game theory and calculates each input variable’s contribution to the model output. This decomposes the overall prediction into a linear combination of input variables, rendering the internal structure of the model more interpretable.

By clearly and numerically presenting the extent to which each feature contributes to a prediction, *SHAP* is highly effective in comparing and analysing changes in feature importance before and after transfer learning. Unlike model-agnostic methods, *SHAP* provides both global and local explanations of the impact of each feature on a specific prediction. It is based on a locally interpretable linear model that expresses predictions as a linear combination of binary variables. This representation allows a quantitative understanding of how the model responds to different combinations of input variables. The *SHAP* explanatory model is defined by the following generalised equation:

(1) $$g\left(z^{\prime }\right)=\;\phi_{0}+\;\sum_{\left\{i=1\right\}}^{M}\phi_{i}z_{i}^{\prime }.$$

Here, $g\left(z^{\prime }\right)$ represents the predicted value of the *SHAP* explanation model (i.e., the local approximation of the original model f), and M denotes the total number of features. The vector $z^{\prime }$ is a binary masking vector, where each element $z_{i}^{\prime }\in \{0,\;1\}$ indicates whether the corresponding feature is included (1) or excluded (0). Additionally, $\emptyset_{0}$ represents the baseline value (i.e., the average prediction), and $\emptyset_{0}$ denotes the Shapley value for feature i. Each $\emptyset_{i}$ is defined as the average marginal contribution of that feature across all possible combinations of feature subsets, by comparing the model’s output when the feature is included versus when it is excluded. Unlike traditional variable importance analysis, this linear model-based *SHAP* approach enables a more precise interpretation of each feature’s contribution within the context of the model’s prediction. In this study, the *SHAP* explanatory model was applied to predictive models both before and after transfer learning, thereby enabling a systematic comparison of how the relative importance of each factor shifts owing to domain differences between farms [26].

## 3. Results

### 3.1. Comparison of Environmental Variable Distributions and Characteristics by Facility

**Figure 7:**
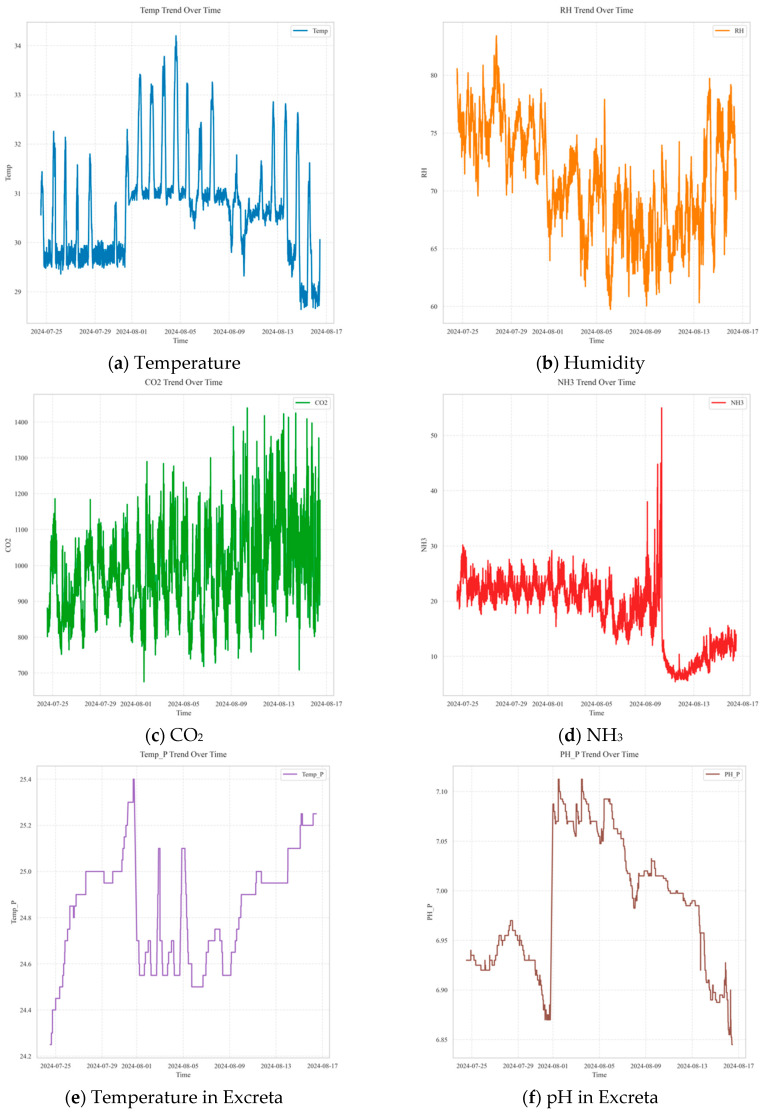

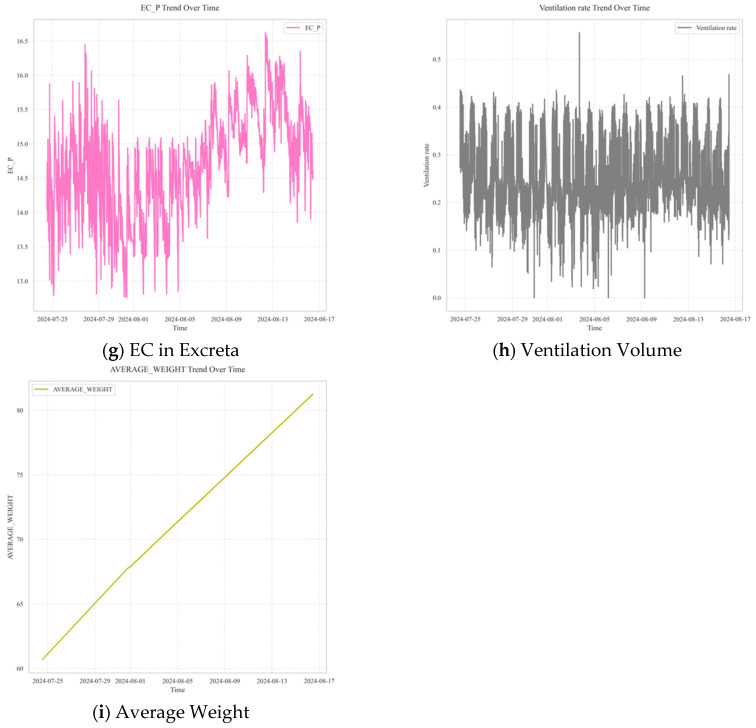
Visualization of data collection for pre-trained model development.

The statistical values and standard deviations of each variable used in the development of the pre-trained model are presented in Table 5, and the overall distributions and temporal variation patterns are shown in Figure 7. During the experimental period, the measured indoor temperature reached a daytime maximum of 34.2 °C and dropped to a nighttime minimum of 27.0 °C, with an average of 30.1 °C. The average RH was 71%, ranging from a maximum of 82% to a minimum of 58%, showing a gradual decline throughout the observation period. This pattern was interpreted as a result of interactions with external climatic conditions. For NH_3_, concentrations ranged from a minimum of 5.22 ppm to a maximum of 55 ppm, with an average of 14.42 ppm, generally remaining within the recommended threshold of 25 ppm. The average CO_2_ concentration was 1011.5 ppm, with fluctuations between 675.6 ppm and 1722.8 ppm.

**Figure 8:**
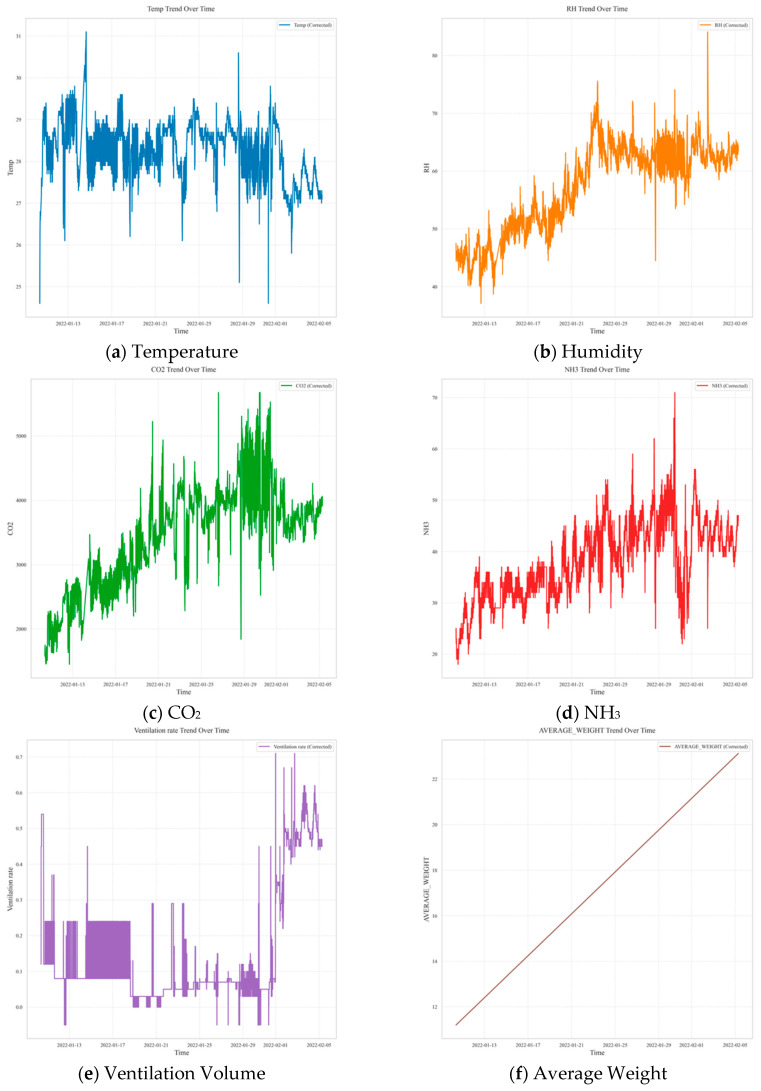
Visualization of data collection for transfer learning model development.

The training data for the transfer learning model are summarised in Table 6 and illustrated in Figure 8. In this dataset, the indoor temperature ranged from 24.6 °C to 31.1 °C, with an average of 28.2 °C. The RH averaged 57.1%, with a maximum of 84.1% and a minimum of 37.1%, indicating slightly lower values than in the source domain. NH_3_ concentrations ranged from 18 to 71 ppm, with an average of 38.1 ppm. CO_2_ concentrations showed significant variability, from 1450 ppm to 5677 ppm, with an average of 3384.1 ppm. These results can be attributed to factors such as higher stocking density and seasonal outdoor climatic conditions.

Fluctuations in CO_2_ and NH_3_ concentrations are interpreted as a consequence of ventilation system operations, wherein ventilation rates are adjusted in response to changes in indoor temperature. In both facilities, a common trend was observed: as temperature increased, ventilation rates increased, resulting in decreased NH_3_ and CO_2_ concentrations. The data confirmed a clear inverse relationship between indoor temperature and ventilation rate, with gas concentrations exhibiting similar regulatory patterns.

Although differences in environmental conditions—such as temperature, RH, and gas concentrations—existed between the datasets used for pre-training and transfer learning, the underlying operational mechanisms, including ventilation strategies, rearing environment variables, and measurement parameters, were consistently maintained. These shared dynamics support the feasibility of applying a transfer-learning-based predictive model to both domains. Therefore, rather than focusing solely on the differences in variable distributions, this study validates the legitimacy of transfer learning by emphasising the similarity in interaction patterns between environmental variables in both settings.

### 3.2. Influence of Input Variables on the Predictive Model

**Figure 9:**
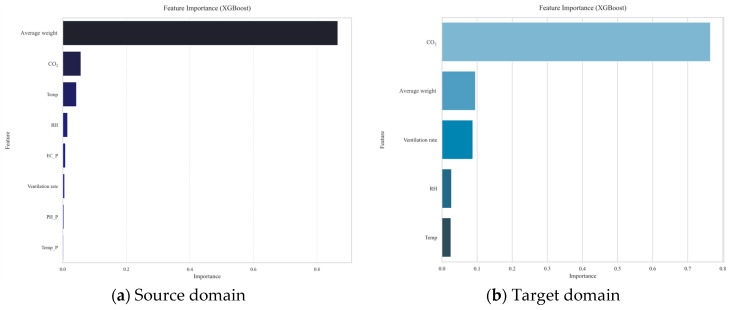
Feature importance: source versus target domain.

Figure 9 presents the results of evaluating and visualising the importance of key variables influencing NH_3_ concentration using data collected from each facility. The analysis revealed that the two most influential factors were the average body weight of the pigs and the CO_2_ concentration, which ranked first and second in feature importance, respectively.

The importance of average body weight can be interpreted as reflecting the production stage of the herd and the associated increase in nitrogen input and excretion. As pigs grow, feed intake and excretion rates generally increase, leading to a greater manure nitrogen load and, consequently, a higher potential for NH_3_ generation at manure and floor surfaces. Therefore, body weight primarily represents a source-term driver linked to the emission potential rather than short-term ventilation dynamics. Since feed intake and manure nitrogen content were not directly measured, this interpretation is presented as a proxy-based explanation grounded in established husbandry relationships.

In contrast, CO_2_ concentration primarily captures ventilation and dilution conditions within the building. While CO_2_ is generated by animal respiration, its indoor concentration is strongly modulated by the effectiveness of air exchange; thus, CO_2_ can serve as an operational proxy for the balance between pollutant accumulation and removal. Under typical management, ventilation is adjusted in response to thermal loads, which affects both CO_2_ and NH_3_ concentrations. This explains the similar temporal patterns observed for CO_2_ and NH_3_ (Figure 7c,d and Figure 8d) and supports the consistently high importance of CO_2_ in both the source and target domains. In the target domain, CO_2_ may become relatively more informative because differences in housing scale and ventilation operation can make short-term NH_3_ variability more strongly governed by ventilation-driven dilution, thereby strengthening the CO_2_–NH_3_ relationship learned by the model.

Notably, despite differences between facilities, average body weight and CO_2_ represent two fundamental and broadly transferable drivers, likely explaining their dominance across both domains. Other predictors regulate NH_3_ predictions through complementary pathways. Ventilation rate directly affects indoor NH_3_ via dilution and removal and also mediates the influence of thermal conditions. Temperature may influence NH_3_ volatilisation and mass transfer from emitting surfaces and can indirectly affect concentrations through ventilation control strategies. Relative humidity may reflect moisture-related conditions of manure and floor surfaces, which can modify emission and transfer processes; however, these effects can be non-linear and site-dependent due to interactions with management practices and ventilation. 

Although this analysis enabled the identification of the relative importance of key variables, it was limited in its ability to quantitatively interpret non-linear interactions and complex interdependencies between the variables. For a more sophisticated interpretation, we therefore additionally applied a model interpretation technique, *SHAP*, as described in Section 4.2.

### 3.3. Performance Evaluation of the Pre-Trained Model

Model validation was performed by splitting the collected dataset into 80% for training and 20% for testing. Predictive performance was evaluated using three metrics: *R*^2^, *RMSE*, and *MAPE*. The pre-trained model demonstrated overall high prediction accuracy, achieving *R*^2^ = 0.96, *RMSE* = 1.22, and *MAPE* = 4.90 (Table 7).

**Figure 10:**
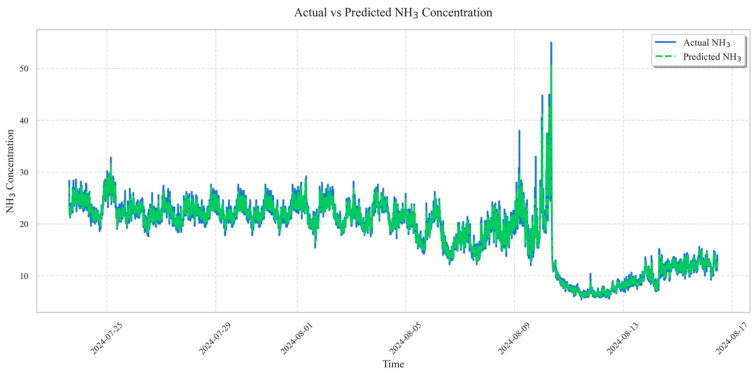
Visualization of prediction performance for pre-trained model.

Figure 10 presents a visualised time-series comparison between the predicted NH_3_ concentrations and the actual measured values. A significant alignment was observed between the two, with the predicted values reliably following the periodicity and patterns of the empirical data. These results indicate that the model possesses sufficient reliability to serve as a foundation for transfer learning.

Notably, the fact that the model demonstrated high generalisability despite being trained on a limited amount of data collected under livestock farming conditions supports its potential to maintain effective performance during subsequent transfers to the target domain. Accordingly, this pre-trained model was employed as the base model for transfer learning experiments using data from an actual pig farm located in Suncheon, to assess whether predictive accuracy could be sustained under new environmental conditions.

### 3.4. Comparison of Predictive Performance: Standalone Model Versus Transfer-Learning-Based Model

**Figure 11:**
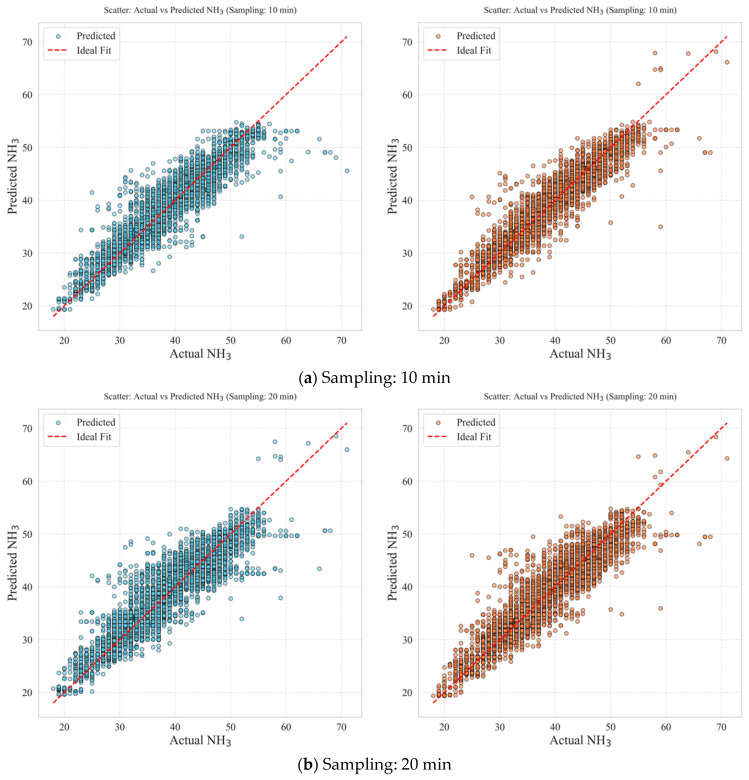

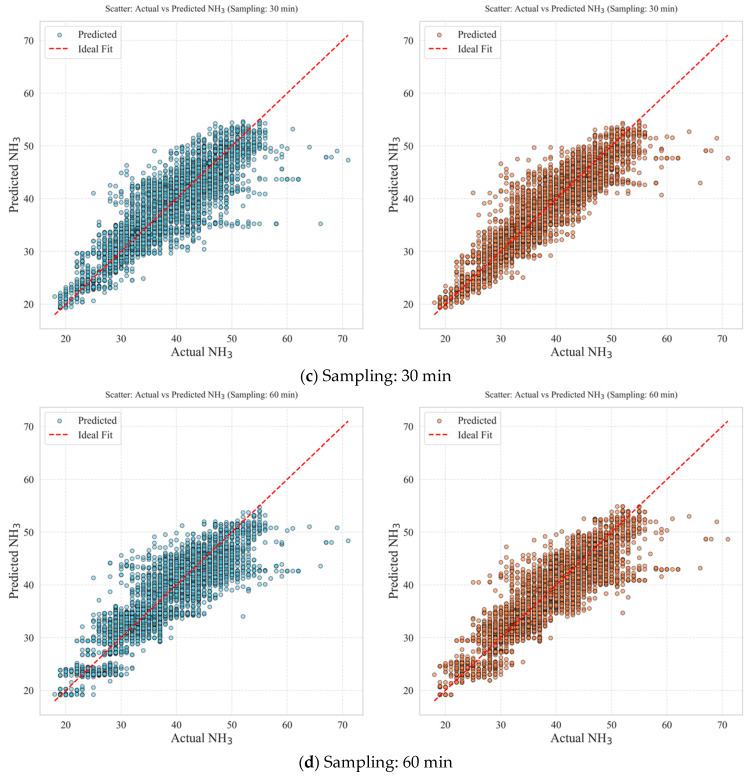
Comparison of prediction performance: transfer learning versus standalone model.

To evaluate the predictive performance of NH_3_ concentration in the target domain, a comparative experiment was designed based on two variables: data collection intervals (10, 20, 30, and 60 min) and training strategy (standalone versus transfer learning). In particular, the analysis focused on how the transfer learning approach, which leverages generalised representations from a pre-trained model, affects prediction accuracy under varying data collection conditions. The experimental results are presented in Table 8, Table 9 and Table 10 and Figure 11. Overall, the transfer-learning-based model (Case B) outperformed the standalone model (Case A) under all conditions. The standalone model, trained solely on target domain data, exhibited a general tendency for predictive performance to improve with shorter data collection intervals. For instance, at the 10 min interval, it achieved relatively good performance with *R*^2^ = 0.79, *RMSE* = 3.45, and *MAPE* = 6.12%. However, when the interval was extended to 30 min, performance decreased to *R*^2^ = 0.67, *RMSE* = 4.03, and *MAPE* = 7.79%. This drop is interpreted as a result of the model’s inability to capture short-term fluctuations in NH_3_ concentration at longer sampling intervals. Interestingly, at the 60 min interval, the model showed partial recovery, likely because long-term patterns and correlations between input variables became more prominent at coarser time resolutions. Nevertheless, the performance of the standalone model remained sensitive to data collection frequency and dataset structure, revealing inherent limitations in its generalisability.

In contrast, the transfer learning model (Case B) leveraged representations from the pre-trained source domain model and was fine-tuned using a subset of data from the target domain. Across all data collection intervals, Case B consistently demonstrated higher predictive accuracy than Case A. Notably, under conditions where the standalone model (Case A) showed diminished performance—such as at the 30 min interval—Case B achieved superior results, with *R*^2^ = 0.85, *RMSE* = 3.31, and *MAPE* = 5.24%. These findings suggest that transfer learning is an effective strategy for maintaining prediction stability and accuracy even in environments with low data resolution or limited sample sizes. Furthermore, by reusing high-level feature representations learned by the pre-trained model, the transfer learning approach maintained excellent performance with shorter training times and lower computational demand, even when the input data were scarce.

To minimise overfitting during fine-tuning, model complexity was controlled using XGBoost regularisation and tree-growth constraints, and model adaptation was carried out using a chronological split to prevent information leakage. Specifically, the model was fine-tuned using the initial segment of the target-domain time series, and final performance was evaluated on an independent evaluation set that was not used for training. Both models exhibited improved predictive performance with shorter data collection intervals; however, the sensitivity to interval changes was notably lower in Case B. In other words, the transfer-learning-based model was less affected by variations in data-sampling frequency and exhibited more consistent accuracy. This consistency indicates that the model internalised the generalised environmental features from the source domain, enabling it to learn effectively from limited data in the target domain.

The above experimental results indicate that the standalone model, which is based on the specific characteristics of an individual domain, responds sensitively to variations in data collection conditions and environmental variables. Consequently, such models may face limitations in maintaining stable performance when applied in real-world settings. In contrast, the transfer-learning-based model demonstrated stable predictive accuracy across a range of data collection conditions and showed strong potential for effective application in actual livestock environments, particularly where data collection is limited or environmental conditions differ substantially. These findings suggest that the transfer learning approach proposed in this study holds significant value for enhancing the generalisability and practical applicability of predictive models in livestock environmental contexts.

### 3.5. SHAP-Based Feature Importance Results

To quantitatively assess the effectiveness of transfer learning and analyse the impact of the pre-trained model on NH_3_ concentration prediction in the target domain, an additional analysis using *SHAP* values was conducted. Two XGBoost-based predictive models were compared: a standalone model trained solely on the target domain data, and a transfer learning model fine-tuned on the same dataset using a model pre-trained on the source domain. *SHAP* values were computed using the *SHAP* Python package (version 0.49.1).

A *SHAP* dot plot was used for comparison, displaying the distribution of *SHAP* values for each input variable across varying input levels. This allowed intuitive interpretation of the influence of each independent variable on NH_3_ concentration. Comparing the two models revealed how transfer learning altered the relative contributions and inference patterns of input variables, enabling improved interpretability and insight into how the model responded to environmental variables.

**Figure 12:**
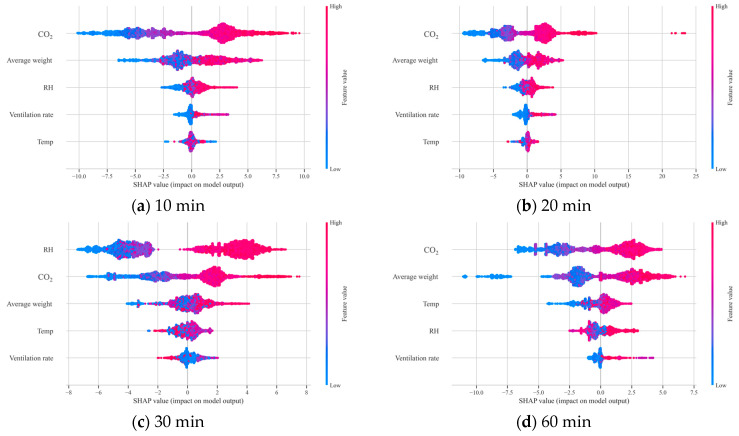
*SHAP* value plot of the standalone model in the target domain.

Figure 12 presents the *SHAP* results from the standalone model, highlighting the feature importance of each variable in predicting NH_3_ concentration. For most data collection intervals (10, 20, and 60 min), CO_2_ concentration emerged as the most influential variable, with *SHAP* values increasing in accordance with input level.

In contrast, for the 30 min interval, RH recorded the highest *SHAP* values and became the dominant variable in the model.

**Figure 13:**
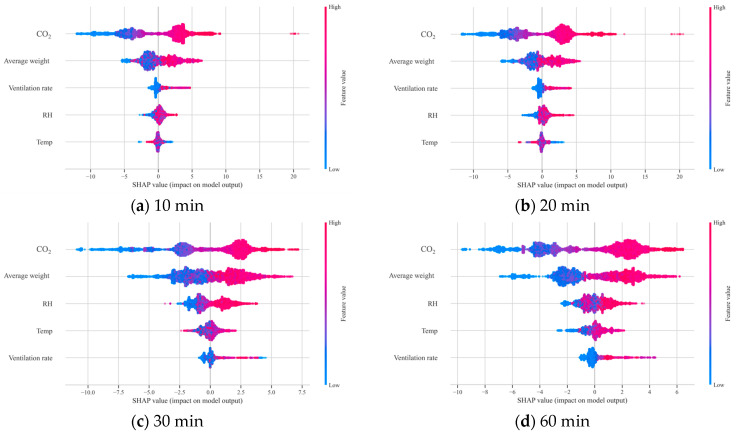
*SHAP* value plot of the pre-trained model after fine-tuning in the target domain.

Figure 13 shows the *SHAP* results from the transfer learning model. Across all intervals, CO_2_ remained the most influential predictor, exhibiting the largest spread of *SHAP* values compared with the other input variables, which indicates its dominant global contribution to NH_3_ prediction under the target-farm conditions. Moreover, relative to the standalone model, the transfer learning model showed a broader range of CO_2_-attributed *SHAP* values, suggesting that CO_2_ captured a wider set of ventilation- and dilution-related operating states after fine-tuning. For instance, at the 30 min interval, the *SHAP* value range for CO_2_ was [−6.8, 7.5] in the standalone model but [−10.8, 7.0] in the transfer learning model. In addition, the *SHAP* dot plots indicate that higher CO_2_ levels generally corresponded to more positive *SHAP* contributions, consistent with elevated NH_3_ under reduced ventilation conditions, whereas lower CO_2_ levels tended to contribute negatively. Overall, these patterns support the interpretation that fine-tuning with pre-learned knowledge reduced over-reliance on any single variable and enabled better reflection of latent patterns within the target domain.

Moreover, while the top-ranked variables remained largely consistent, differences between their *SHAP* values were reduced after applying transfer learning, leading to fewer fluctuations in the relative importance rankings.

## 4. Discussion

### 4.1. Interpretation of SHAP-Based Feature Importance

Based on the *SHAP* results presented in Section 3.5 (Figure 12 and Figure 13), we interpret the key drivers of NH_3_ prediction and discuss their practical implications.

From a management perspective, the consistently high importance of CO_2_ indicates that it can be used as a practical proxy for ventilation adequacy. Therefore, a simple CO_2_ linked control can be applied, in which fan operation is increased when CO_2_ persistently exceeds a farm-defined target band, and the system is inspected for ventilation faults if CO_2_ remains high despite control actions. Because optimal setpoints depend on housing type and season, these thresholds should be calibrated to each farm’s operating range. This aligns with previous studies [20,26], indicating that CO_2_ is a key indicator of ventilation rates and temperature, and thus, critical in explaining NH_3_ variation.

This deviated from previous findings and suggests that RH may have been disproportionately weighted under this specific condition, potentially distorting model interpretability. Indeed, model performance under this interval was lower than that for other intervals (Table 7), suggesting that coarse sampling resolutions and altered variable interactions can adversely affect training.

This suggests that fine-tuning with pre-learned knowledge reduced over-reliance on any single variable and enabled better reflection of latent patterns within the target domain. As a result, the transfer learning model produced more consistent predictions even when the sampling interval was changed, which explains its more stable performance across varying sampling intervals. This indicates that the transfer learning model incorporated the specific characteristics of the target domain more effectively and learned variable interactions more precisely.

In conclusion, the *SHAP* analysis confirmed that the transfer learning model formed a more generalised structure of feature importance and maintained stable prediction performance even under coarse sampling conditions. In real-world applications where data quality or sampling conditions vary, transfer learning, by leveraging the embedded knowledge of the pre-trained model, can serve as a practical and robust alternative for environmental prediction in livestock systems.

### 4.2. Applicability Boundaries and Future Optimisation

The proposed framework is designed for situations where NH_3_-labelled data in the target facility are limited. In practice, even when the source and target farms differ in breeding scale, breed, or diet, the model can be re-trained or fine-tuned if sufficient target-domain NH_3_-labelled data are available to represent the local operating conditions. However, when only a small amount of target-labelled data is available, larger differences between domains may require more local calibration and may reduce the benefit of transfer learning. This is because changes in growth stage, feeding practice, and manure characteristics can alter the NH_3_ emission potential and its relationship with ventilation-driven dilution. A limitation of this study is that the target-domain evaluation was conducted using data from a single commercial farm; therefore, the generalisability of the proposed framework to other farms and operating conditions requires further validation. Therefore, future work will investigate how much target-domain data are needed as domain differences increase and will develop more robust adaptation approaches, such as multi-farm pre-training and simple monitoring of model reliability during deployment.

In addition, detailed optimisation of the hardware configuration and computational efficiency is beyond the scope of this work. However, on an Apple silicon-based CPU platform (Apple M4, 16 GB unified memory), the end-to-end execution of the proposed workflow required approximately 16 s per run, including data loading, preprocessing (resampling and imputation), model inference, and *SHAP* value computation. The model inference step itself required only a small fraction of the total runtime, whereas the *SHAP* computation accounted for the majority of the processing time. Given that predictions were generated at 10–60 min intervals, this runtime supports the practical feasibility of periodic on-farm monitoring on commodity CPU hardware. The deployment pipeline consists of sensor acquisition/data logging → quality control and resampling/imputation → XGBoost inference → *SHAP* interpretation → dashboard/alert output.

## 5. Conclusions

This study investigated the scalability of data-driven models for predicting ammonia (NH_3_) concentration across pig farming facilities with different climatic and structural conditions and proposed a transfer-learning-based approach. An XGBoost model was pre-trained using high-resolution data collected at a testbed pig house at Kongju National University and then adapted to a commercial farm in Suncheon. The results show that the transfer learning model achieved consistently improved prediction performance compared with a standalone model trained only on target-farm data and maintained stable accuracy under limited target-domain data across multiple sampling intervals. *SHAP*-based interpretation further indicated that the transfer learning model provided more consistent explanatory patterns across key variables, supporting the interpretability of the model under target-farm conditions.

Overall, these findings suggest that transfer learning can support accurate and robust NH_3_ prediction in practical livestock environments where sensor deployment and long-term data collection are constrained. The proposed framework may serve as a foundation for smart livestock housing systems and cross-farm decision-support tools. Future research will extend validation to additional farms and operating conditions and integrate the model with dispersion modelling to quantify NH_3_ transport within and around pig houses, thereby supporting early-warning applications for odour management and improved environmental sustainability.

## Figures and Tables

**Table 1:** Quantitative comparison of source- and target-domain facilities.

	Source Domain	Target Domain
Number of pigs	24 pigs	900 pigs
Production stage	Fattening pigs	Growing pigs
Growing period	16–20 weeks of age	7–10 weeks in the growing house
Average weight	59.77 → 81.25 kg	7 → 25 kg
Ventilation system	Mechanical ventilation with wall-mounted exhaust fans	Mechanical ventilation with chimney-type exhaust fans

**Table 2:** Scope and methods of data collection for pre-trained model development.

Category	Detailed Items	Measurement Method
Environmental Information	Temperature	Automatic
	RH	
	CO_2_	
	NH_3_	
	Ventilation Volume	
Excreta Information	Temperature in Excreta	Automatic
	pH in Excreta	
	EC in Excreta	
Individual Management	Average Weight	Automatic/Manual

Abbreviations: electrical conductivity (EC). Note: NH_3_ concentration was measured during the campaign but used only as the prediction target (label); it was not included among the input features.

**Table 3:** Scope and methods of data collection for transfer learning model development.

Category	Detailed Items	Measurement Method
Environmental Information	Temperature	Automatic
	RH	
	CO_2_	
	NH_3_	
	Ventilation Volume	
Individual Management	Average Weight	Automatic/Manual

Note: NH_3_ concentration was measured during the campaign but used only as the prediction target (label); it was not included among the input features.

**Table 4:** Hyperparameters of the pre-trained model.

Algorithms	Hyperparameter	Defined Values
XGBoost	max_depth	10
	learning_rate	0.1
	n_estimators	100
	subsample	0.8

Abbreviations: extreme gradient boosting (XGBoost).

**Table 5:** Maximum, minimum, and mean values of the collected data for pre-trained model development.

Detailed Items	Average	Maximum	Minimum	Standard Deviation
Temperature	30.1 °C	34.2 °C	27 °C	1.21 °C
RH	71.17%	82.28%	58.24%	4.94%
CO_2_	1011.52 ppm	1722.8 ppm	675.6 ppm	132.89 ppm
NH_3_	14.42 ppm	55 ppm	5.2 ppm	6.13 ppm
Ventilation Volume	0.13 min^−1^	0.55 min^−1^	0 min^−1^	0.09 min^−1^
Temperature in Excreta	25.4 °C	27.6 °C	22.7 °C	0.87 °C
pH in Excreta	6.96	7.11	6.81	0.13
EC in Excreta	15.29	17.43	12.42	1.045
Average Weight	103.91 kg	111.74 kg	96.07 kg	9.94 kg

Abbreviations: electrical conductivity (EC).

**Table 6:** Maximum, minimum, and mean values of the collected data for transfer learning model development.

Detailed Items	Average	Maximum	Minimum	Standard Deviation
Temperature	28.2 °C	31.1 °C	24.6 °C	0.69 °C
RH	57.1%	84.1%	37.1%	7.67%
CO_2_	3384.1 ppm	5677 ppm	1450 ppm	767.78 ppm
NH_3_	38.1 ppm	71 ppm	18 ppm	7.46 ppm
Ventilation Volume	0.1 min^−1^	0.71 min^−1^	0 min^−1^	0.16 min^−1^
Average Weight	17.1 kg	23.1 kg	11.1 kg	3.44 kg

**Table 7:** Performance evaluation of the pre-trained model.

Model Algorithm	*R* ^2^	*RMSE*	*MAPE*
XGBoost	0.96 (0.954–0.967)	1.22 (1.090–1.314)	4.90% (4.58–4.99)

Abbreviations: Values are reported as point estimates (95% CI).

**Table 8:** Case A: Prediction performance comparison by data sampling interval in the target facility.

Evaluation Metric	Data Sampling Interval (min)			
	**10**	**20**	**30**	**60**
*R* ^2^	0.80 (0.737–0.899)	0.76 (0.638–0.860)	0.67 (0.537–0.759)	0.76 (0.714–0.823)
*RMSE*	3.28 (2.229–4.171)	3.41 (2.631–4.277)	4.03 (3.529–4.573)	3.68 (2.129–4.345)
*MAPE*	5.79% (4.54–6.39)	6.98% (5.65–8.42)	7.79% (5.54–8.89)	8.13% (5.63–8.79)

Abbreviations: coefficient of determination (*R*^2^), mean absolute percentage error (*MAPE*), root mean square error (*RMSE*). Values are reported as point estimates (95% CI).

**Table 9:** Case B: Prediction performance comparison by data sampling interval in the target facility using transfer learning.

Evaluation Metric	Data Sampling Interval (min)			
	**10**	**20**	**30**	**60**
*R* ^2^	0.91 (0.905–0.917)	0.87 (0.875–0.889)	0.85 (0.857–0.879)	0.80 (0.786–0.816)
*RMSE*	2.19 (2.153–2.299)	2.63 (2.580–2,734)	2.83 (2.782–2.998)	3.31 (3.203–3.436)
*MAPE*	4.07% (4.04–4.24)	4.96% (4.87–5.10)	5.24% (5.15–5.41)	6.29% (6.23–6.59)

Abbreviations: coefficient of determination (*R*^2^), mean absolute percentage error (*MAPE*), root mean square error (*RMSE*). Values are reported as point estimates (95% CI).

**Table 10:** Pre- and post-transfer learning prediction performance comparison.

Evaluation Metric	Data Sampling Interval (min)			
	**10**	**20**	**30**	**60**
Δ*R*^2^	0.11	0.11	0.18	0.04
Δ*RMSE*	−1.09	−0.78	−1.2	−0.37
Δ*MAPE*	−1.72%	−2.02%	−2.55%	−1.84%

Abbreviations: coefficient of determination (*R*^2^), mean absolute percentage error (*MAPE*), root mean square error (*RMSE*).

## Data Availability

The datasets generated and analysed during the current study are available from the corresponding author upon reasonable request.
